# A facile approach to nanoarchitectured three-dimensional graphene-based Li–Mn–O composite as high-power cathodes for Li-ion batteries

**DOI:** 10.3762/bjnano.3.59

**Published:** 2012-07-17

**Authors:** Wenyu Zhang, Yi Zeng, Chen Xu, Ni Xiao, Yiben Gao, Lain-Jong Li, Xiaodong Chen, Huey Hoon Hng, Qingyu Yan

**Affiliations:** 1 School of Materials Science and Engineering, Nanyang Technological University, Singapore 639798, Singapore; 2 TUM CREATE Research Centre @ NTU, Nanyang Technological University, Singapore 637459, Singapore; 3 Energy Research Institute @ NTU, Nanyang Technological University, Singapore 637553, Singapore; 4 School of Civil and Environmental Engineering, Nanyang Technological University, Singapore 639798, Singapore; 5 Research Center for Applied Sciences, Academia Sinica, Taipei 11529, Taiwan

**Keywords:** cathode, graphene, Li-ion battery, lithium manganate

## Abstract

We report a facile method to prepare a nanoarchitectured lithium manganate/graphene (LMO/G) hybrid as a positive electrode for Li-ion batteries. The Mn_2_O_3_/graphene hybrid is synthesized by exfoliation of graphene sheets and deposition of Mn_2_O_3_ in a one-step electrochemical process, which is followed by lithiation in a molten salt reaction. There are several advantages of using the LMO/G as cathodes in Li-ion batteries: (1) the LMO/G electrode shows high specific capacities at high gravimetric current densities with excellent cycling stability, e.g., 84 mAh·g^−1^ during the 500th cycle at a discharge current density of 5625 mA·g^−1^ (~38.01 C capacity rating) in the voltage window of 3–4.5 V; (2) the LMO/G hybrid can buffer the Jahn–Teller effect, which depicts excellent Li storage properties at high current densities within a wider voltage window of 2–4.5 V, e.g., 93 mAh·g^−1^ during the 300th cycle at a discharge current density of 5625 mA·g^−1^ (~38.01 C). The wider operation voltage window can lead to increased theoretical capacity, e.g., 148 mAh·g^−1^ between 3 and 4.5 V and 296 mAh·g^−1^ between 2 and 4.5 V; (3) more importantly, it is found that the attachment of LMO onto graphene can help to reduce the dissolution of Mn^2+^ into the electrolyte, as indicated by the inductively coupled plasma (ICP) measurements, and which is mainly attributed to the large specific surface area of the graphene sheets.

## Introduction

Lithium-ion batteries (LIBs) are considered the primary candidate as the power source for plug-in and hybrid electric vehicles [1]. Although LiCoO_2_ is widely used as a commercial cathode for Li-ion batteries, there are several drawbacks, including high cost and toxicity. Spinel Lithium Manganate (LMO) is a promising cathode material for rechargeable Li-ion batteries due to the high energy density, improved operating safety, low cost and low toxicity [2]. However, the low electrical conductivity (10^−6^ S·cm^−1^) and poor cycling performance of LMO are the main issues associated with this material [3–4]. The poor cyclability is attributed to the collapse of the crystal structure caused by (1) Mn^2+^ (formed through Mn^3+^ disproportionation) dissolution into the electrolyte, (2) the electrolyte decomposition and (3) the Jahn–Teller effect [5], which refers to the large volume changes due to the transition from the cubic to the tetragonal spinel phase during the charge/discharge cycling. This results in large distortion of the crystal structure and causes severe capacity loss.

In order to solve these issues, many methods have been implemented. In general, these methods can be divided into two categories: chemical doping and microstructure modification. The chemical doping method involves elevating the average manganese ion oxidation state so as to decrease the amount of Mn^3+^ in LMO, e.g., by replacing manganese with monovalent [6] or multivalent cations [7–9]. However, doping into LMO tends to reduce the practical capacity of the material. On the other hand, promising progress in the modification of micro/nanostructures of LMO has been made [10–13]. Ordered mesoporous LMO, created by using silica templates, favors rapid kinetics for both lithium ions and electron transport, which show excellent cyclability between 3 and 4.3 V but with relatively low capacities <100 mAh·g^−1^ at 1 C rate (capacity rating) [14]. The silica template used in this synthesis process has to be removed by an additional step, which may introduce impurities to LMO. In order to suppress the dissolution of manganese ions and reduce the electrical resistivity, coating LMO powders with another functional layer [3–4,15–16] can help. However, to achieve a uniform functional layer on the LMO powder surface still remains a technical challenge.

The fabrication of nanoarchitectured 3D electrodes is a promising approach to improve the power density and cyclability of LIBs [17–18]. Basically, such a strategy is based on the design of a nanostructured, metal current collector, by using Cu or Al nanorods to form a 3D conducting scaffold, to improve the kinetics of Li diffusion and electron transfer in the electrode. As a material with high electrical conductivity and large surface area [19–22], graphene has attracted much attention for battery electrode applications. The hybrids of transition-metal-oxide nanocrystals attached onto graphene sheets have shown much improvement of LIB anode performance [23–26], in which charge transfer is improved and the agglomeration of the metal oxide nanocrystals is prevented. Moreover, a recent report [27] indicated that by combining nanolayer carbon (e.g., graphene or nanoporous carbon) with sulfide anode may help to solve the issue of the dissolution of Li_2_S into the electrolyte during cycling. This could be considered as a useful strategy to improve the dissolution of Mn^2+^ from the LMO cathode. However, the preparation of graphene-based cathodes is still limited [24,28]. A possible reason may be that many methods for the preparation of cathode materials involve high-temperature reactions that may destroy the graphene structure. Although a graphene-based cathode may be difficult to prepare, the conducting scaffold formed by graphene sheets is ideal for the formation of 3D architectured cathodes to improve the performance of LIB further, especially at high C rates.

Currently, the reported synthesis of graphene-based electrode materials for LIB is mostly based on the method of Hummers [23–26,29–32], which requires the presynthesis of graphene oxides (GOs) and post treatments on the GOs to improve the electrical conductivity. There are also some reports on the preparation of LIB anodes using graphene prepared by chemical vapor deposition [33–34]. However, such application will be limited due to cost and scalability.

Electrochemical exfoliation is a newly developed method that can be used to prepare highly conductive graphene sheets in high yield and at low cost [35]. Moreover, it is also known that inorganic redox-active materials can be electrochemically deposited at low cost and by means of simple processes. It is thus attractive to integrate these two approaches together in an electrochemical process to synthesize graphene-based electrode materials, especially for LIB cathodes. In this paper, we report a facile approach to synthesize lithium manganate/graphene (LMO/G) hybrids by combining the exfoliation of graphene sheets with the deposition of Mn_2_O_3_ nanowalls in a one-step electrochemical process, followed by molten salt lithiation to convert Mn_2_O_3_ to LMO. The molten salt process [36–37] requires lower temperature and shorter reaction time than that of conventional solid-state synthesis, which is useful to preserve the structure of the graphene sheets. The as-prepared LMO in the LMO/G hybrids are nanocrystals with sizes in the range of 3–10 nm, whereas the weight ratio between the LMO and graphene can be readily tailored by changing the concentration of MnSO_4_ in the electrochemical process. The LMO/G hybrid cathode shows excellent Li storage properties under fast lithium intercalation/deintercalation processes. The optimized cathode delivers a stable specific capacity of ~84 mAh·g^−1^ during the 500th cycle at a gravimetric current density of 5625 mA·g^−1^ (~38.01 C) with a voltage window of 3–4.5 V, which corresponds to a specific power density of 100 kW·kg^−1^ and a specific energy density of 278 Wh·kg^−1^. Also, the LMO/G hybrid can buffer the Jahn–Teller effect [5], which leads to improved Li storage properties over a wider voltage range of 2–4.5 V, e.g., 93 mAh·g^−1^ during the 300th cycle at a discharge current density of 5625 mA·g^−1^ (~38.01 C). Last and most important, it is found that the dissolution of Mn^2+^ into the electrolyte is very much reduced for the LMO/G sample as compared to that of commercially purchased LMO, as indicated by the inductively coupled plasma (ICP) study. This is important for achieving a stable performance of the LMO-based cathodes. The methodology proposed here should also be applicable for the preparation of other lithium-metal-oxide/graphene hybrids for high-power LIB applications.

## Results and Discussion

**Figure 1:**
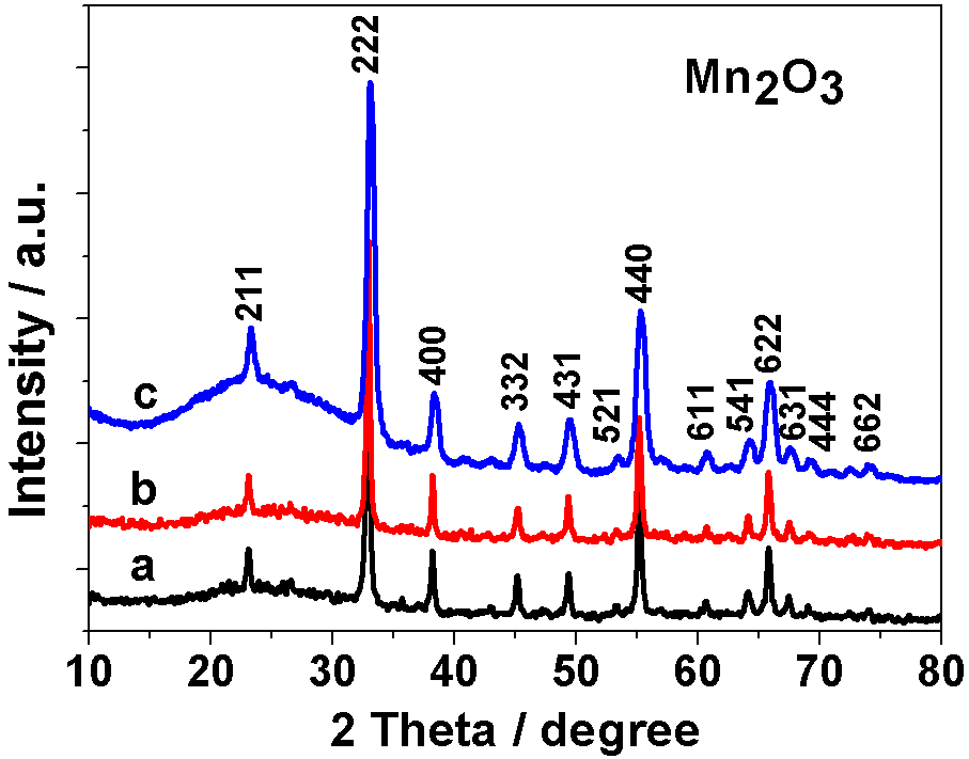
Powder XRD patterns of the Mn_2_O_3_/graphene hybrids prepared by an electrochemical method with various concentrations of MnSO_4_: (a) 0.15, (b) 0.3 and (c) 0.6 M.

The samples from the electrochemical deposition were prepared by the electrolysis of MnSO_4_ using graphite sheets as the working electrodes. In this process, metal-oxide deposition and graphene exfoliation are implemented simultaneously. The XRD patterns (see Figure 1) confirm the formation of a cubic Mn_2_O_3_ (JCPDS No. 41-1442) phase in the various samples prepared, by changing the concentration of MnSO_4_. The grain sizes of Mn_2_O_3_ in the various samples are estimated to be about 20 nm, based on the full width at half maximum of the diffraction peaks by using Scherrer’s equation. The bump at around 2θ = 25° is attributed to the glass holder. There are no other detectable peaks corresponding to impurity phases in the XRD patterns.

**Figure 2:**
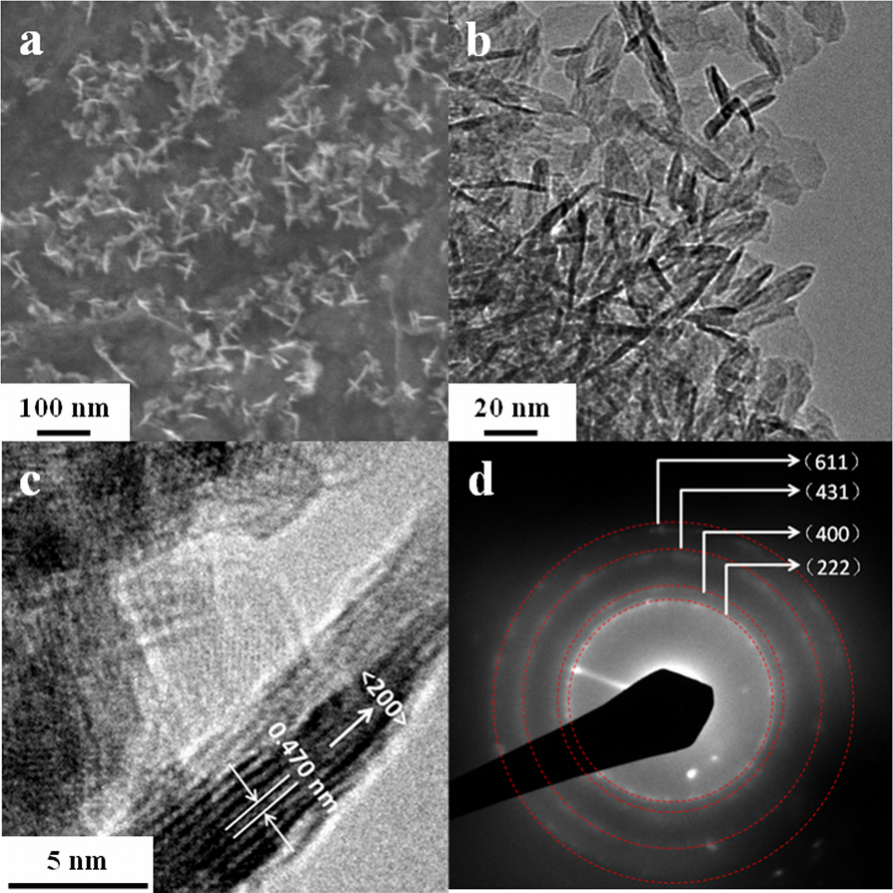
(a) SEM, (b) TEM, (c) HRTEM images, and (d) SAED of Mn_2_O_3_/graphene prepared with 0.15 M MnSO_4_.

The SEM and TEM images of the as-prepared samples are shown in Figure 2. For the sample prepared with a MnSO_4_ molar concentration of 0.15 M, the SEM image (see Figure 2a) shows that the nanowalls are uniform and are attached onto the surface of the nanosheets. These nanowalls are mostly vertically aligned with respect to the surface of the nanosheets. The corresponding TEM image (see Figure 2b) reveals that these nanowalls are 3–5 nm in thickness and 25–30 nm in diameter. The high-resolution TEM (HRTEM) image (Figure 2c) and the selected-area electron diffraction pattern (Figure 2d) confirms the formation of the cubic Mn_2_O_3_ phase. The observed interlattice spacing of 0.470 nm in the HRTEM corresponds to the (200) planes of the cubic Mn_2_O_3_ (JCPDS No. 41-1442) phase.

**Figure 3:**
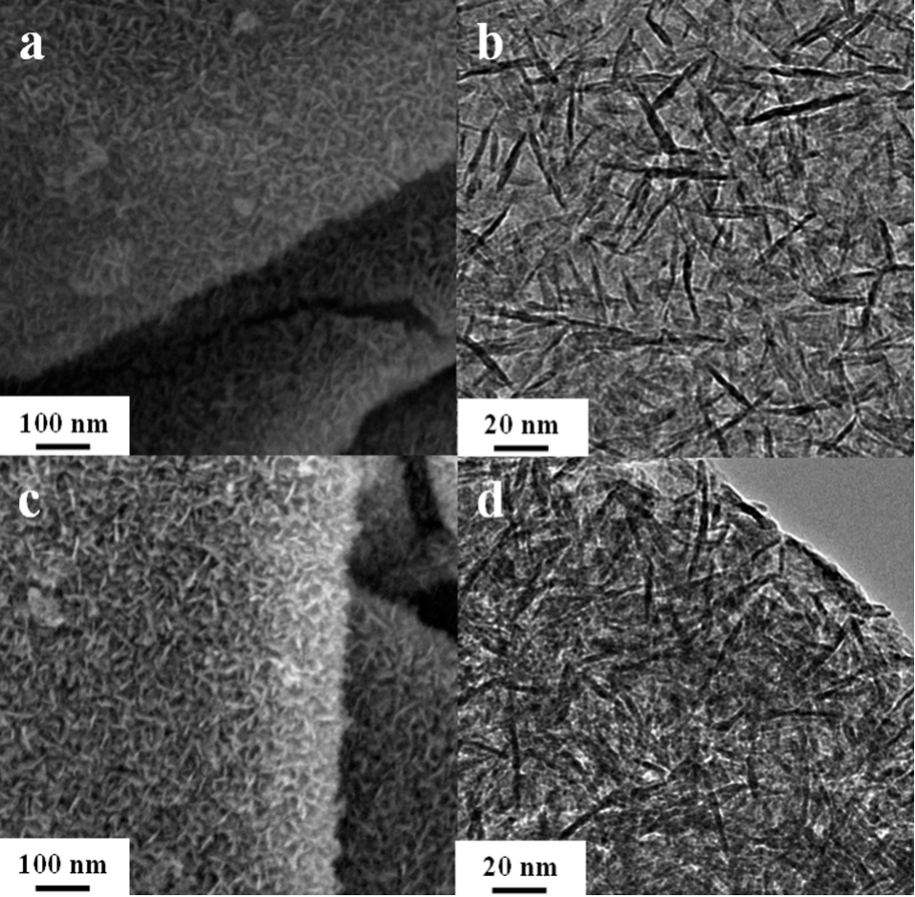
(a, c) SEM and (b, d) TEM images of Mn_2_O_3_/graphene prepared with (a, b) I_MO:G_ = 1.34, (c, d) I_MO:G_ = 2.57.

The formation of graphene is confirmed using Raman spectroscopy (see Supporting Information File 1, Figure S1a). The Raman spectra of the samples show that the intensity ratio I_2D/G_ of the 2D band (located at 2720 cm^−1^) to the G band (located at 1580 cm^−1^) is 0.16, which is similar to that reported for electrochemically exfoliated graphene [35]. The weight ratios, I_MO:G_, of Mn_2_O_3_:graphene are found to be directly related to the concentration of MnSO_4_ in the precursors. The thermal gravimetric analysis (TGA) results (see Supporting Information File 1, Figure S2a) show that the I_MO:G_ are 0.82, 1.34 and 2.57 for samples prepared with MnSO_4_ molar concentrations of 0.15, 0.3 and 0.6 M, respectively. For Mn_2_O_3_/graphene samples with higher I_MO:G_ values, the shape of Mn_2_O_3_ remains in the form of nanowalls, while the density of the Mn_2_O_3_ nanowalls on the graphene sheets increases (see Figure 3a–d).

The above observation indicates that the entire preparation process of Mn_2_O_3_/G samples can be divided into two parts. The first part is the exfoliation of the graphene sheets. In this part, the electrostatic force drives the SO_4_^2−^ ions into the spacing between the carbon layers of the graphite electrode and breaks the connection between graphene layers. The second part is the deposition of manganese oxide. In this part, Mn^2+^ is oxidized and deposited onto the graphene surface during the exfoliation process of the graphene sheets.

**Figure 4:**
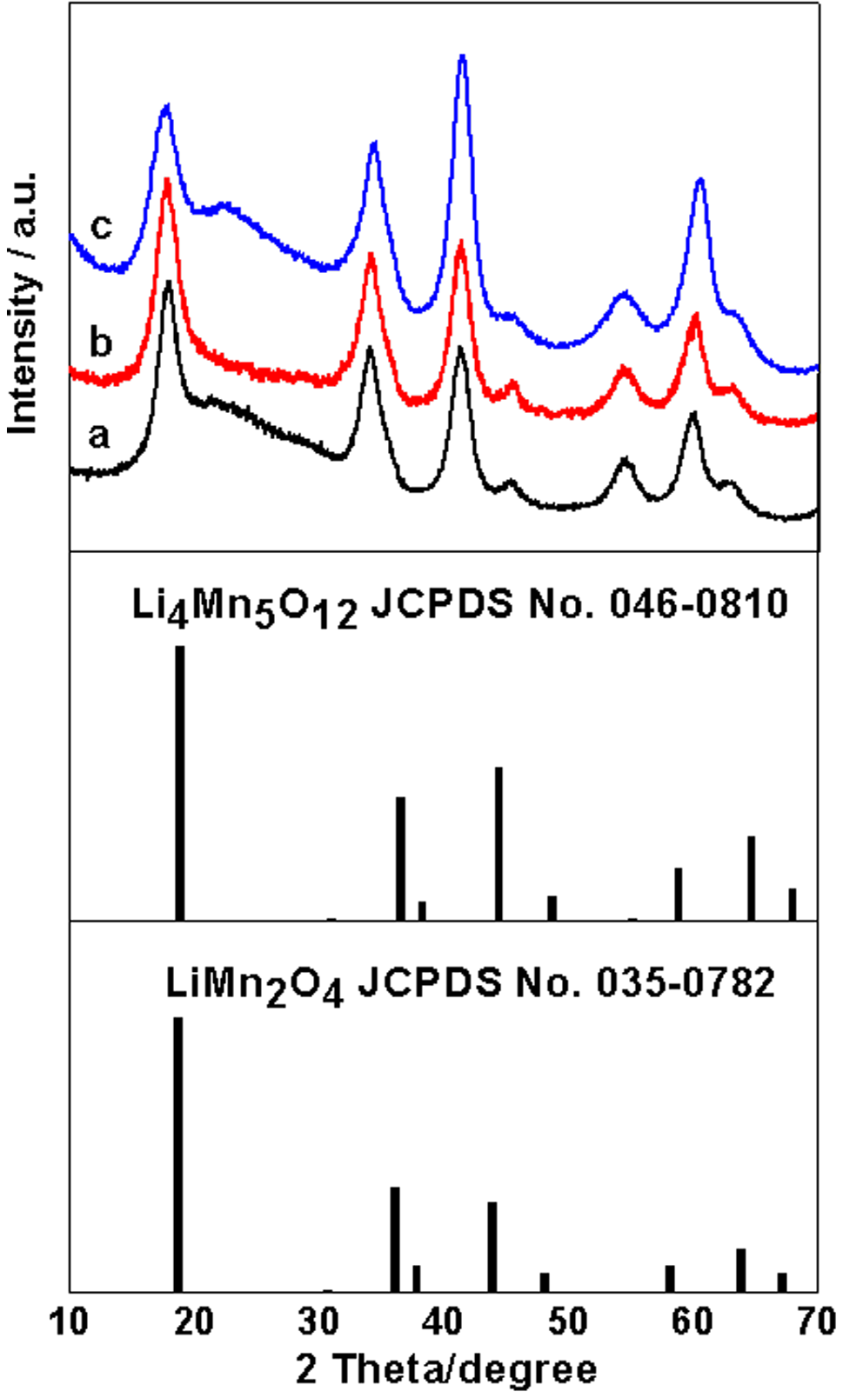
Powder XRD patterns for lithiated Mn_2_O_3_/graphene with (a) I_MO:G_ = 0.82, (b) I_MO:G_ = 1.34, (c) I_MO:G_ = 2.57 by molten salt reaction at 300 °C for 2 h in Ar.

The as-prepared Mn_2_O_3_/G samples with different I_MO:G_ values are lithiated in a molten salt reaction [36] under Ar atmosphere, which is followed by annealing in Ar gas environment at *T* = 623 K for 30 min. The XRD patterns (see Figure 4) of the sample after the lithiation and annealing process confirm the formation of the cubic spinel LiMn_2_O_4_ phase (JCPDS No. 35-0782). Here, the determination of the exact phase of the sample after the lithiation and annealing process is based on both the XRD analysis and inductively coupled plasma (ICP) measurements. The XRD pattern of cubic spinel LiMn_2_O_4_ (JCPDS No. 35-0782) is similar to that of cubic spinel Li_4_Mn_5_O_12_ (JCPDS No. 046-0810). The ICP results (see Table 1) indicate that the molar ratios of Li:Mn are 0.495, 0.49 and 0.502, respectively, for the different Mn_2_O_3_/G samples (e.g., I_MO:G_ = 0.82, 1.34 and 2.57) after the lithiation and annealing process. These ICP results are approximately the ideal ratios for LiMn_2_O_4_, which confirms the stoichiometry of the final samples. The grain size of the spinel LiMn_2_O_4_ (LMO) is estimated to be only 3.2 nm, based on the full width at half maximum of the diffraction peaks using Scherrer’s equation. This grain size is much smaller than that of Mn_2_O_3_, which is possibly due to a recrystallization process. No impurity phase is detectable using XRD.

**Table 1:** ICP results of LiMn_2_O_4_/graphene hybrids with I_LMO:G_ = 1.22, 2.00 and 4.49.

I_LMO:G_	1.22	2.00	4.49	Ideal

Li/Mn	0.495	0.49	0.502	0.5

After the lithiation process, the TGA results (see Supporting Information File 1, Figure S2b) indicate that the weight ratios, I_LMO:G_, between the LMO and graphene are 1.22, 2.00 and 4.49 for the samples prepared by lithiating Mn_2_O_3_/G samples with I_MO:G_ = 0.82, 1.34 and 2.57, respectively. Here, the calculated I_LMO:G_ values are 1.07, 1.90, and 4.71 for Mn_2_O_3_/G samples with I_MnO:G_ values of 0.82, 1.34 and 2.57, respectively, which are close to the measured I_LMO:G_ values.

The Raman spectra show that the intensity ratio of the 2D band to the G band (see Supporting Information File 1, Figure S1b) is 0.20 for LMO/G samples after annealing in an Ar/H_2_ atmosphere. This suggests that the structure of the graphene sheets is not significantly disrupted after the lithiation and annealing process. However, it is worth noting that the intensity ratio between the D band (located at 1360 cm^−1^) and G band increases after the lithiation process, which indicates the increased number of defects in the graphene sheets.

**Figure 5:**
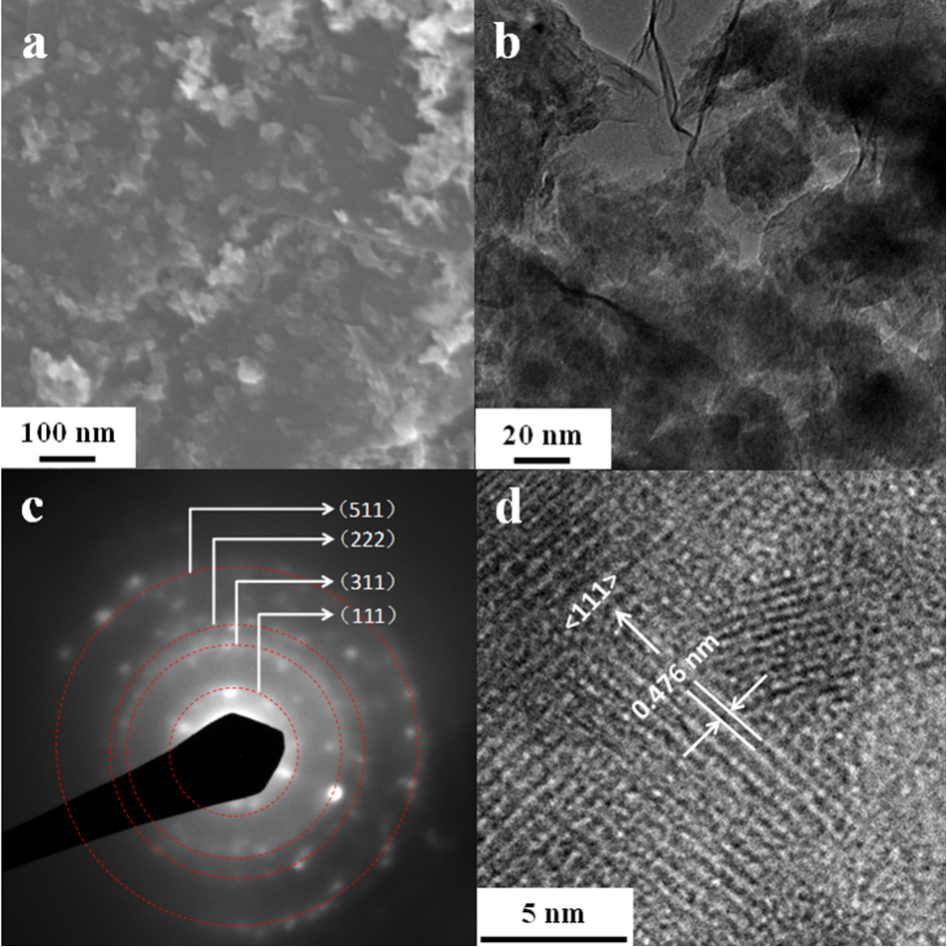
(a) SEM, (b) TEM, (c) SAED and (d) HRTEM images of LiMn_2_O_4_/graphene hybrids prepared from Mn_2_O_3_/graphene (I_MO:G_ = 0.82) by molten salt reaction at 300 °C for 2 h in argon.

For the LMO/G sample with I_LMO:G_ = 1.22, the SEM image (see Figure 5a) shows that the Mn_2_O_3_ nanoplates change to nanoparticles after the lithiation process, but they are still attached onto the graphene sheets. The size of the particles is in the range of 20–30 nm as revealed from TEM observation (see Figure 5b). The SAED pattern (see Figure 5c) obtained for these nanoparticles indicates that they are cubic LiMn_2_O_4_ (JCPDS No. 35-0782), which is consistent with the XRD results. The HRTEM image (see Figure 5d) shows that these LiMn_2_O_4_ nanoparticles are polycrystalline with grain sizes in the range of 3–10 nm. The observed interlattice spacing of 0.476 nm in the HRTEM corresponds to the (111) planes of the cubic spinel LiMn_2_O_4_ phase (JCPDS No. 35-0782).

For the LMO/G samples with higher I_LMO:G_ values, the SEM images (see Supporting Information File 1, Figure S3a,b) show similar morphology to that of the sample with I_LMO:G_ = 1.22, except that the loading of the LMO particles on the graphene sheets is higher. The HRTEM images (see Supporting Information File 1, Figure S3c,d) show that the grain sizes of LMO in the samples with higher I_LMO:G_ = 2.00 and 4.49, are also in the range of 3–10 nm.

**Figure 6:**
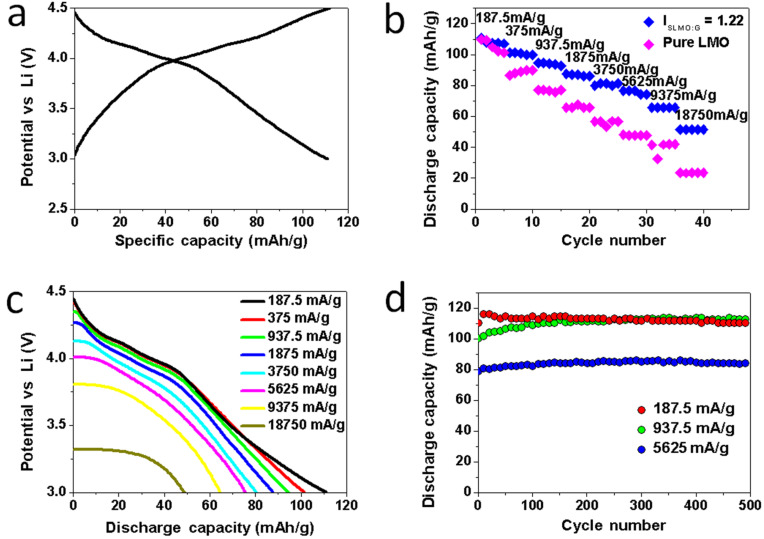
(a) The second-cycle voltage profiles of LiMn_2_O_4_/graphene (I_LMO:G_ = 1.22) between 3 and 4.5 V at 1.27 C. (b) Rate response of commercial LiMn_2_O_4_ and LiMn_2_O_4_/graphene (I_LMO:G_ = 1.22) between 3 and 4.5 V. (c) Discharge voltage profiles of LiMn_2_O_4_/graphene (I_LMO:G_ = 1.22) between 3 and 4.5V. (d) Cycling performance of LiMn_2_O_4_/graphene (I_LMO:G_ = 1.22) at 187.5 mA·g^−1^ (1.27 C), 937.5 mA·g^−1^ (6.33 C) and 5625 mA·g^−1^ (38.01 C) discharge rates. The specific capacity was calculated based on the mass of active material.

To evaluate the cathode performance of the LMO/G hybrids, half cells were fabricated based on a Swagelok configuration [38–40]. Figure 6a shows the representative discharge/charge voltage profiles of the LMO/G hybrid with I_LMO:G_ = 1.22, named as LMO/G (I_LMO:G_ = 1.22), for the second cycle at 1.27 C rate (187.5 mA·g^−1^) between 3 and 4.5 V. The 4 V plateau during discharge corresponds to the reduction of Mn^4+^ to Mn^3.5+^ through the reaction of Li_1−_*_x_*Mn_2_O_4_ + *x*e^−^ + *x*Li^+^ → LiMn_2_O_4_, where *x* is ~1 [41]. The highly reversible lithium insertion/extraction process is indicated by the symmetric nature of the charge and discharge curves. The sloping plateau is attributed to the low-temperature synthesis process [36].

The rate capability of the LMO/G (I_LMO:G_ = 1.22) electrode is evaluated under different discharge currents between 3 and 4.5 V. Figure 6b shows that the LMO/G (I_LMO:G_ = 1.22) electrode delivered second-cycle discharge capacities of 108, 101, 94.5, 87, 81, 76.5, 65.5 and 51.5 mAh·g^−1^ at current densities of 187.5 (1.27 C), 375 (2.53 C), 937.5 (6.33 C), 1875 (12.67 C), 3750 (25.34 C), 5625 (38.01 C), 9375 (63.34 C) and 18750 (126.68 C) mA·g^−1^, respectively. These values correspond to a series of energy densities of 402, 378, 353, 323, 299, 278, 236 and 175 Wh·kg^−1^ at different power densities of 3.37, 6.80, 17.17, 34.20, 67.28, 100.08, 163.39 and 315.00 kW·kg^−1^, respectively. The LMO/G electrodes (I_LMO:G_ = 1.22) present higher energy densities at high power densities than porous LMO (282 Wh·kg^−1^ at 15 kW·kg^−1^) [42], porous LMO in aqueous electrolytes (110 Wh·kg^−1^ at 10 kW·kg^−1^) [43], single crystalline LMO nanowires [41] (243 Wh·kg^−1^ at 54 kW·kg^−1^) and LMO nanotubes (304 Wh·kg^−1^ at 5.6 kW·kg^−1^) [44].

For the purpose of comparison, the rate capability of commercially purchased pure LMO powders was also investigated between 3 and 4.5 V (see Figure 6b). The corresponding SEM image reveals that the pure LMO powder is 0.3–1 µm in size (see Supporting Information File 1, Figure S4), while the XRD pattern and ICP measurements confirm the high purity of the cubic spinel phase (JCPDS No. 35-0782). The discharge capacity of pure LMO fades rapidly with increasing current densities (see Figure 6b) and exhibits second-cycle discharge capacities of 109.5, 88, 77, 66, 57, 48, 32 and 23 mAh·g^−1^ at current densities of 187.5 (1.27 C), 375 (2.53 C), 937.5 (6.33 C), 1875 (12.67 C), 3750 (25.34 C), 5625 (38.01 C), 9375 (63.34 C) and 18750 (126.68 C) mA·g^−1^, respectively. These values correspond to a series of energy densities of 382, 310, 282, 254, 226, 194, 155 and 117 Wh·kg^−1^ at different power densities of 3.17, 6.36, 16.07, 32.69, 62.05, 85.59, 104.57 and 134.72 kW·kg^−1^, respectively. The commercially purchased pure LMO powder shows comparable discharge capacities to that of LMO/G electrodes at low current densities, e.g., <3 C. However, at high current densities, e.g., >25 C, the LMO/G (I_LMO:G_ = 1.22) electrodes show much higher discharge capacities than that of pure LMO, which indicates that the conducting scaffold of graphene sheets improves the kinetics of Li ion diffusion and electron transfer.

The voltage profiles of the LMO/G (I_LMO:G_ = 1.22) electrode under various discharge currents are presented in Figure 6c, which shows that the discharge voltages are maintained at >3.3 V even at a very high current density of 18750 mA·g^−1^.

The cycling performance of the LMO/G (I_LMO:G_ = 1.22) electrode is evaluated (see Figure 6d) at current densities of 187.5 mA·g^−1^ (1.27 C), 937.5 mA·g^−1^ (6.33 C) and 5625 mA·g^−1^ (38.01 C) in the voltage range of 3–4.5 V. When discharging at 1.27 C and 6.33 C, the LMO/G (I_LMO:G_ = 1.22) electrode delivered discharge capacities of 110 mAh·g^−1^ and 100 mAh·g^−1^, respectively, during the second cycle, which did not show any obvious decrease and remained at 110 mAh·g^−1^ and 100 mAh·g^−1^, respectively, during the 500th cycle with capacity retentions of 100% per cycle. Even at a very high discharging rate of 38.01 C, it exhibited highly reversible specific capacities of 79 mAh·g^−1^ during the second cycle and 84 mAh·g^−1^ during the 500th cycle. The cycling performance of LMO/G with I_LMO:G_ = 2.00 and 4.49 are also evaluated at 1 C in the voltage range of 3–4.5 V (see Supporting Information File 1, Figure S5). With second-discharge capacities of 91–101 mAh·g^−1^, the LMO/G electrodes with higher I_LMO:G_ values are able to deliver highly reversible specific capacities of 82–93 mAh·g^−1^ during the 500th cycle.

**Figure 7:**
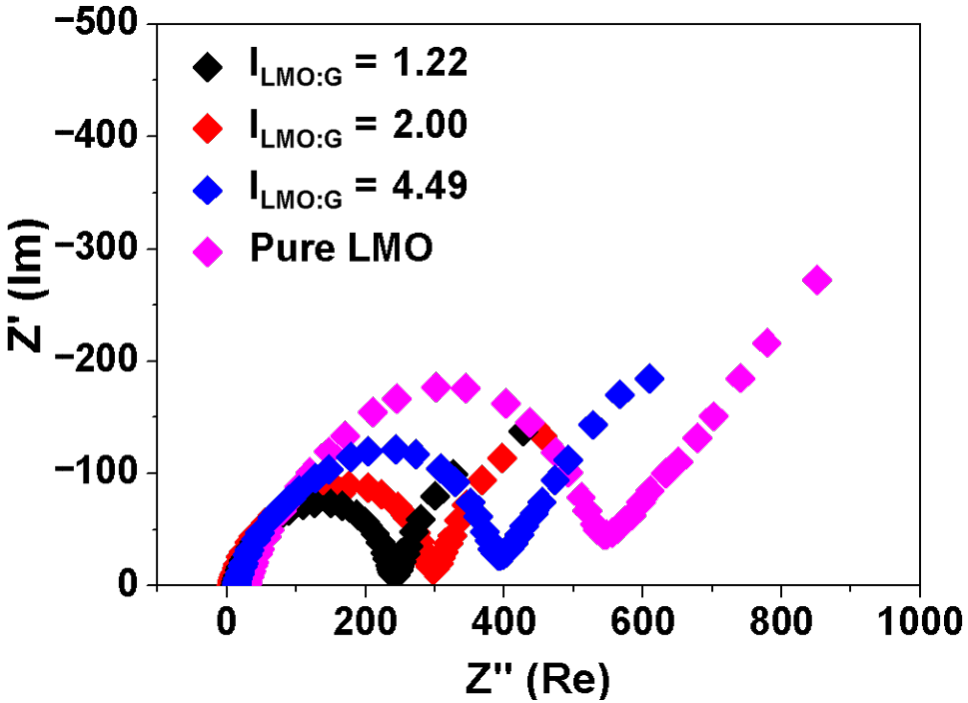
The Nyquist plots of LiMn_2_O_4_/graphene and pure LiMn_2_O_4_ electrodes at the fifth fully discharged state.

The good Li storage properties of the LMO/G hybrids at high current densities are supported by the impedance studies. The electrodes at the fifth fully discharged state are tested under identical conditions. Figure 7 shows the Nyquist plots of the LMO/G and pure LMO electrodes. The pure LMO electrode displays a larger semicircular diameter than that of LMO/G electrodes, and thereby indicates a poorer charge-transfer conductance [29]. The diameters of the semicircles are also smaller for samples with lower I_LMO:G_ values, which suggests that the graphene sheets serve as the 3D conducting scaffold to improve the Li storage performance of the hybrid electrodes at high current densities.

**Figure 8:**
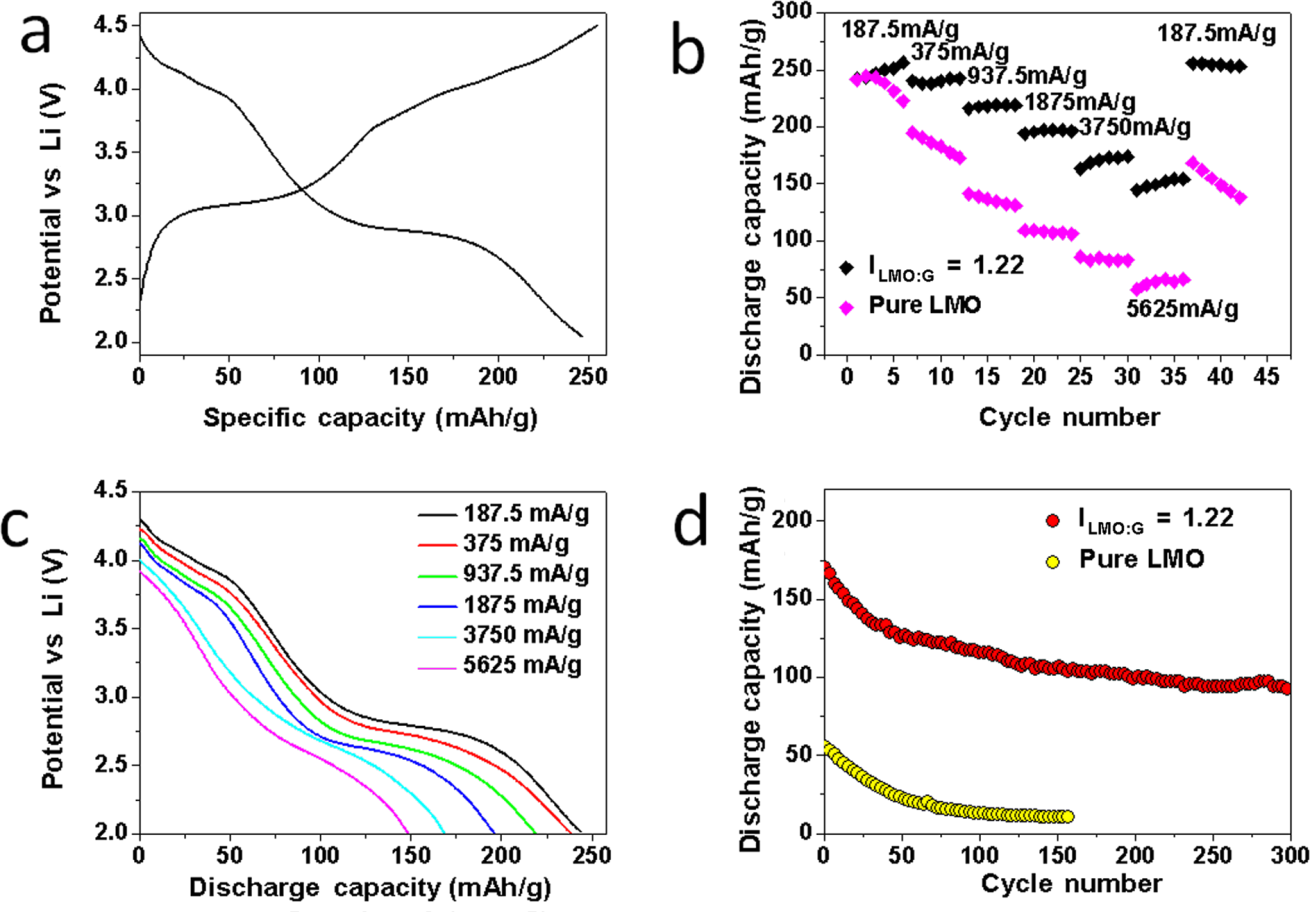
(a) The second-cycle discharge voltage profiles of LiMn_2_O_4_/graphene (I_LMO:G_ = 1.22) between 2 and 4.5 V at 1.27 C. (b) Rate response of commercial LiMn_2_O_4_ and LiMn_2_O_4_/graphene (I_LMO:G_ = 1.22) between 2 and 4.5 V. All samples were cycled at the same discharge/charge current densities. (c) Discharge voltage profiles of LiMn_2_O_4_/graphene (I_LMO:G_ = 1.22) between 2 and 4.5 V. (d) Cycling performance of LiMn_2_O_4_/graphene (I_LMO:G_ = 1.22) at 5625 mA·g^−1^ (38.01 C) discharge current and 2812 mA·g^−1^ (19.00 C) charge current.

It is known that the spinel LMO phase undergoes an additional redox reaction at 3 V, which increases the theoretical capacity, e.g., 148 mAh·g^−1^ in a voltage range of 3–4.5 V and 296 mAh·g^−1^ in a voltage range of 2–4.5 V. However, when discharged between 2 and 4.5 V, the LMO cathode suffers from the Jahn–Teller effect with a large volume change due to the transition from cubic to tetragonal spinel [45], which results in a large distortion of the crystal structure and causes severe capacity loss. In the LMO/G hybrids, the flexible graphene sheets may help to buffer the strain of the crystal. To test this, we examined the cathode performance of the LMO/G samples in the voltage range between 2 and 4.5 V. Figure 8a shows the representative discharge/charge voltage profiles of the LMO/G (I_LMO:G_ = 1.22) for the second cycle at 1.27 C rate (187.5 mA·g^−1^) between 2 and 4.5 V. The plateau at ~3 V corresponds to the further reduction of Mn^3.5+^ to Mn^3+^ through LiMn_2_O_4_ + *y*e^−^ + *y*Li^+^ → Li_1+_*_y_*Mn_2_O_4_, where *y* is ~1 [46]. The symmetrical feature of the charge and discharge curves indicates the highly reversible lithium insertion/extraction process.

The rate capability of the LMO/G (I_LMO:G_ = 1.22) electrode was evaluated under the same charge/discharge currents between 2 to 4.5 V. Figure 8b shows that the LMO/G (I_LMO:G_ = 1.22) electrode delivers second-cycle discharge capacities of 245, 238, 217, 196, 167 and 148 mAh·g^−1^ at current densities of 187.5 (1.27 C), 375 (2.53 C), 937.5 (6.33 C), 1875 (12.67 C), 3750 (25.34 C) and 5625 mA·g^−1^ (38.01 C), respectively. After cycling at 5625 mA·g^−1^ (38.01 C), the discharge capacity of the LMO/G (I_LMO:G_ = 1.22) electrode recovers back to 257 mAh·g^−1^ when changing the current density to 187.5 mA·g^−1^ (1.27 C), showing a good rate performance. The high C-rate performance is slightly worse for samples with higher I_LMO:G_ values (see Supporting Information File 1, Figure S6). For example, at a current density of 5625 mA·g^−1^ (38.01 C), the LMO/G (I_LMO:G_ = 2.00) and LMO/G (I_LMO:G_ = 4.49) electrodes deliver discharge capacities of 128 and 117 mAh·g^−1^, respectively, during the second cycle.

The voltage profiles corresponding to each current density are plotted in Figure 8c. The plateaus at 4 and 3 V can be clearly differentiated at high current densities (e.g., even at 38.01 C). This indicates that the transition from cubic to tetragonal spinel LiMn_2_O_4_ through the reaction mentioned above can successfully take place even at very high current densities, e.g., 5625 mA·g^−1^, while this transition may not occur easily for bulk LMO [46] at such high current density, mainly due to the large constrain on the structural changes in bulk LMO [10] and LMO with coarsened grains [46].

**Figure 9:**
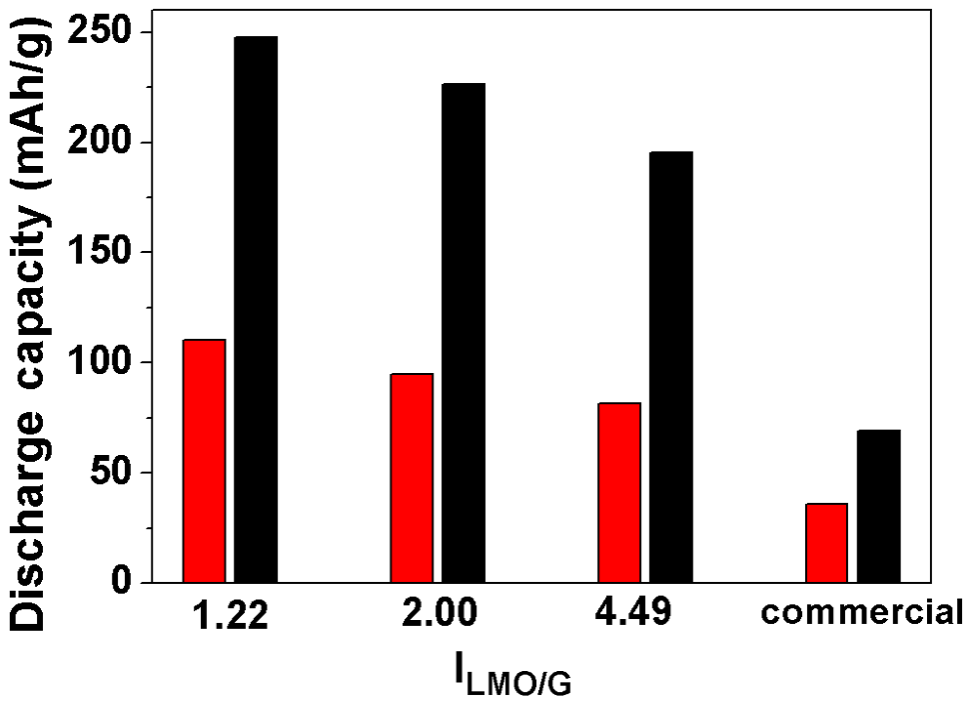
The second-cycle discharge capacities of LiMn_2_O_4_/graphene with various I_LMO:G_ and commercial LiMn_2_O_4_ at 187.5 mA·g^−1^ (1.27 C) discharge current.

The cycling performance of the LMO/G (I_LMO:G_ = 1.22) electrode was also evaluated at very high current densities, e.g., a discharge current density of 5625 mA·g^−1^ (38.01 C) and a charge current density of 2812.5 mA·g^−1^ (19.00 C), between 2 and 4.5 V (see Figure 8d). The LMO/G (I_LMO:G_ = 1.22) electrode depicts a specific capacity of 170 mAh·g^−1^ during the second cycle, which remains at 93 mAh·g^−1^ during the 300th cycle. Based on the voltage profiles under such cycling conditions, the corresponding energy density and power density were calculated to be 284 Wh·kg^−1^ and 17.04 kW·kg^−1^, respectively, during the 300th cycle. Here, it is worth pointing out that the charge rate is chosen as 19.00 C (2812.5 mA·g^−1^) to mimic the fast battery charging process. In fact, some reports on the rate capabilities of cathode materials use a high discharge C rate and a low charge C rate, which are different from the really fast battery charging process, especially for plug-in electric vehicles. For LMO/G electrodes with I_LMO:G_ = 2.00 and 4.49, the cycling responses under similar testing parameters, e.g., a discharge current density of 5625 mA·g^−1^ (38.01 C) and a charge current density of 2812.5 mA·g^−1^ (19.00 C), are also evaluated (see Supporting Information File 1, Figure S7). For example, LMO/G electrodes with I_LMO:G_ = 2.00 delivers a discharge capacity of 150 mAh·g^−1^ during the second cycle and 90 mAh·g^−1^ during the 150th cycle. For LMO/G electrodes with I_LMO:G_ = 4.49, the discharge capacity is 123 mAh·g^−1^ during the second cycle and decreases to 69 mAh·g^−1^ during the 150th cycle. Pure LMO shows much worse cycling response at such high current densities (see Figure 8d). It depicts a discharge capacity of 55 mAh·g^−1^ for the second cycle, which decays rapidly to 10 mAh·g^−1^ during the 150th cycle. The comparison of the cathode performance between the LMO/G samples and pure LMO electrodes is summarized in Figure 9.

For LMO cathode, there is an additional concern about the dissolution of the Mn^2+^ ions into the electrolyte [5]. Thus, the chemical stability associated with manganese dissolution was also explored by ICP measurements. LMO/G hybrids and pure LMO with an identical amount of 100 mg LMO in the samples were immersed in the electrolyte (LiPF_6_ in EC/DEC, 5 mL) at 50 °C for 72 h. The manganese concentrations in the electrolytes are 11.76, 14.22, 17.82 and 23.35 ppm (see Table 2) for LMO/G hybrids with I_LMO:G_ = 1.22, 2.00, 4.49 and pure LMO, respectively. As compared to pure LMO powder, the dissolution of the Mn element into the electrolyte is much reduced for the LMO/G hybrids, especially for samples with higher graphene content (e.g., I_LMO:G_ = 1.22). This is possibly due to the wrapping of graphene sheets onto the LMO nanocrystals and the very large specific surface area of the graphene sheets, e.g., >100 m^2^·g^−1^. Thus, the graphene sheets may act as a physical adsorption layer to anchor the Mn^2+^ ions. Increasing the graphene content in the hybrid sample helps to reduce the dissolution of Mn into the electrolyte further.

**Table 2:** ICP results of Mn dissolution from LiMn_2_O_4_/graphene (LMO/G). LMO/G hybrids and pure LMO with an identical amount of 100 mg LMO in the samples were immersed in the electrolyte (LiPF_6_ in EC/DEC, 5 ml) at 50 °C for 72 hours.

I_LMO:G_	1.22	2.00	4.49	Commercial

Mn concentration (ppm)	11.76	14.22	17.82	23.35

The superior electrochemical performance of LMO/G electrodes is ascribed to three aspects. First, the LMO/G exhibits fast kinetics of Li-ion and electron diffusion, as examined by the electrochemical impedance spectroscopy, which suggests that the 3D conducting scaffold formed by graphene sheets and the fine grain size of the LMO achieved by such a synthesis process help to improve the Li-storage performance of the hybrid electrodes at high current densities. Second, the LMO/G electrodes are demonstrated to show improved capacity retention at high current densities in the voltage range of 2–4.5 V. For the LMO/G hybrids, the fine LMO grains (e.g., 3–10 nm) are embedded in the 3D conducting scaffold formed by the graphene sheets, which can effectively relieve the issue caused by the Jahn–Teller effect, by allowing the LMO grains to expand/contract freely with the support of the conducting graphene sheets. Finally, the incorporation of graphene scaffold is herein proved to be an effective way to suppress Mn dissolution into the electrolyte. In fact, Mn dissolution is one of the biggest concerns for LMO, which significantly affects the cycling stability. It is especially serious for nanosized LMO due to their large surface area exposed to the electrolyte. Mn dissolution causes the collapse of the crystal structure, which leads to the capacity losses during cycling. For LMO/G hybrids, the graphene sheets serve as a physical adsorption layer of anchoring Mn^2+^ cations and thereby enhance the cyclability of the electrodes.

## Conclusion

In conclusion, a novel and facile approach was developed to synthesize a nanoarchitectured LMO/G hybrid as a positive electrode material for lithium-ion batteries. The process combines the deposition of Mn_2_O_3_ and exfoliation of graphite electrodes in a one-step electrochemical process, followed by molten salt lithiation. The weight ratios between the LMO and graphene can be readily adjusted by simply changing the concentration of Mn^2+^ in the electrochemical process. These LMO/G electrodes show excellent cathode performances with high specific capacities and stable cyclability at high current densities in different voltage ranges, e.g., 2–4.5 V and 3–4.5 V. This is mainly due to the several advantages of such hybrids: (1) The combination of the highly conductive 3D graphene scaffold and the small grain size of the LMO (e.g., 3–10 nm) greatly enhance the kinetics of charge transfer in the electrode; (2) the flexible graphene sheets can buffer the volume strain of the LMO grains caused by the transition from cubic to tetragonal spinel and, hence, improve the cycling stability in the voltage range of 2–4.5 V; and (3) the graphene sheets may effectively reduce the dissolution of Mn into the electrolyte and improve the cycling performance.

## Experimental

### Mn_2_O_3_/graphene hybrid synthesis

Mn_2_O_3_ and graphene hybrids were prepared by electrochemical method. Highly oriented pyrolytic graphite sheets (HOPG, 1.5 cm × 1.5 cm × 0.3 mm) and platinum wires (0.2 mm in diameter) were used as the working electrodes and counter electrodes, respectively. The electrolyte was prepared by mixing MnSO_4_, CH_3_COONa and Li_2_SO_4_ with deionized water. The concentrations of Mn^2+^ were selected to be 0.15, 0.3 and 0.6 M. The molar concentrations of CH_3_COONa were equivalent to that of MnSO_4_. The concentrations of Li_2_SO_4_ were 0.85, 0.7 and 0.4 M for the three solutions with 0.15, 0.3 and 0.6 M MnSO_4_. The electrochemical cells were heated to 60 °C and a stepwise voltage of 0–10 V was applied between the working electrode and the counter electrode for 30 min. The products were collected through vacuum filtration, washed several times with deionized water, and centrifuged to remove large graphite particles, followed by drying at 50 °C overnight.

#### Lithiation of Mn_2_O_3_/graphene

The lithiation process was carried out via molten salt reaction. A mixture of 9.9 mg LiNO_3_ was dissolved in deionized water with the addition of 50 mg of as-prepared Mn_2_O_3_/graphene. After vigorous stirring, the solution was dried at 80 °C and then heated to 300 °C in argon gas for 2 h. The resultants were rinsed with deionized water. Thereafter, the final products were annealed at 350 °C for 30 min in argon gas.

#### Materials characterization

Powder XRD patterns were obtained on a Shimadzu XRD-6000 X-ray diffractometer with Cu Kα irradiation. SEM imaging was performed on a JEOL JSM-7600F operating at 5 kV. TEM and HR-TEM images were obtained on a JEOL JEM-2100 operating at 200 kV. Raman spectra were obtained on a WITec CRM 200 with a wavelength of 488 nm and a spot size of 0.5 mm. Thermogravimetry analyses were carried out on TGA Q500 from 298–1073 K at a heating rate of 10 K·min^−1^ in air. The analyses of Li/Mn ratio was performed on inductively coupled plasma atomic emission spectrometer (ICP-AES; PerkinElmer OPTIMA 2000 DV).

Mn dissolution was examined by immersing LMO/G and pure LMO with identical weight of 100 mg LMO in 5 mL LiPF_6_ in EC/DEC at 50 °C for 72 h. The organic solvent was evaporated and the sediments were dissolved in aqueous 4 M HNO_3_, followed by examination of the manganese concentrations with ICP-AES (PerkinElmer OPTIMA 2000 DV). The results were calculated back to the original manganese concentrations in the electrolyte.

#### Electrochemical properties characterization

Electrochemical measurements were carried out on half cells. The electrodes were fabricated by mixing LMO/G (90 wt %) and poly(vinylidene fluoride) (10 wt %) in *N*-methyl-2-pyrrolidone. The mixture was stirred overnight and then cast onto aluminium foils to form uniform electrodes followed by drying in vacuum at 50 °C for 10 h. As for pure LMO electrodes, commercial LMO (80 wt %, purchased from Sigma-Aldrich), Super-P (10 wt %) and poly(vinylidene fluoride) (10 wt %) were mixed and cast onto aluminium foils. CR2032 coin cells were assembled in an argon-filled glove box with the contents of oxygen and moisture below 0.1 ppm, using lithium foils as the counter and reference electrode and 1 M LiPF_6_ in ethylene carbonate and dimethyl carbonate (1:1 v/v) as the electrolyte. All cells were tested on a NEWARE system at 4.5–3 V versus Li^+^/Li. All capacities were calculated based on the mass of LMO and all C-rates were calculated according to the theoretical capacity of 148 mAh·g^−1^. The electrochemical impedance was performed on the aforementioned CR2032 coin cells with lithium foil as the counter and reference electrode. All cells were measured at the fifth fully discharged state. INPHAZE^TM^ EIS system was employed to measure the electrochemical impedance spectra. The amplitude of the alternating voltage was set at 5 mV and the frequency range was 1000 kHz to 10 MHz.

## Supporting Information

Raman spectrum, TGA results, SEM and HRTEM images and electrochemical performance figures.

File 1 Additional figures.
