# Supplementary material for: A facile approach to nanoarchitectured three-dimensional graphene-based Li–Mn–O composite as high-power cathodes for Li-ion batteries
Source: Beilstein J Nanotechnol. 2012 Jul 17;3:513–23. doi: 10.3762/bjnano.3.59 (PMC3458596; doi:10.3762/bjnano.3.59)
Supplement: File 1 — Additional figures. [file Beilstein_J_Nanotechnol-03-513-s001.pdf]

Supporting Information

for

A facile approach to nanoarchitectured three-dimensional graphene-based Li–Mn–O composite as high-power cathodes for Li-ion batteries

Wenyu Zhang[1,2], Yi Zeng[1], Chen Xu[1,3], Ni Xiao[1], Yiben Gao[4], Lain-Jong Li[5], Xiaodong Chen[1], Huey Hoon Hng[1] and Qingyu Yan*[1,2,3]

Address: [1]School of Materials Science and Engineering, Nanyang Technological University, Singapore 639798, Singapore, [2]TUM CREATE Research Centre @ NTU, Nanyang Technological University, Singapore 637459, Singapore, [3]Energy Research Institute @ NTU, Nanyang Technological University, Singapore 637553, Singapore, [4]School of Civil and Environmental Engineering, Nanyang Technological University, Singapore 639798, Singapore and [5]Research Center for Applied Sciences, Academia Sinica, Taipei 11529, Taiwan

Email: Qingyu Yan - alexyan@ntu.edu.sg

* Corresponding author

Additional figures

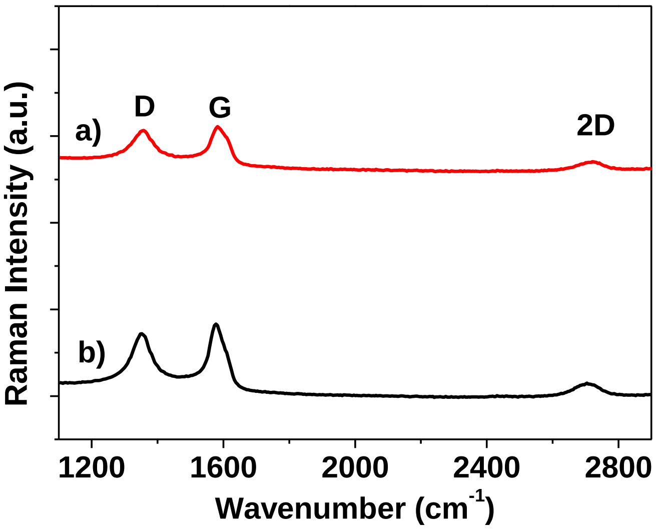

Figure S1: Raman spectra for (a) Mn_2O_3/graphene prepared with 0.15 M $MnSO_4$ in the electrochemical process and (b) $LiMn_2O_4$/graphene prepared by lithiating Mn_2O_3/graphene with $I_{MO:G}$ = 0.99 by molten salt reaction. The D, G and 2D bands are indicated.

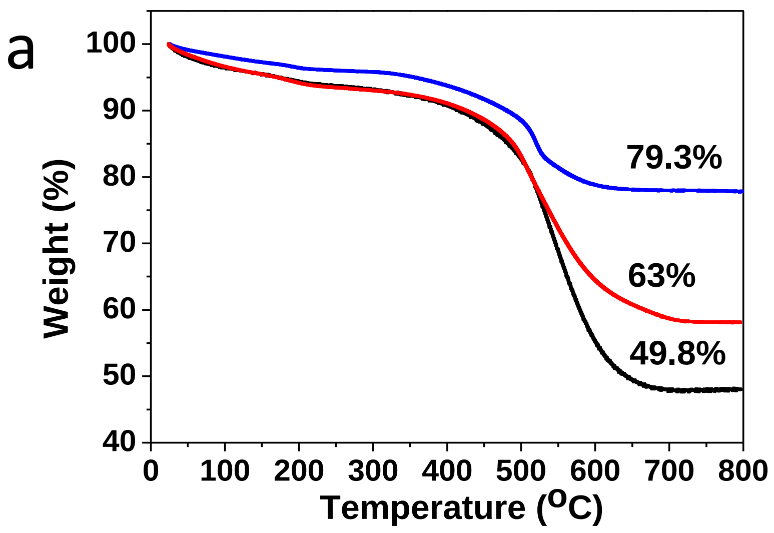

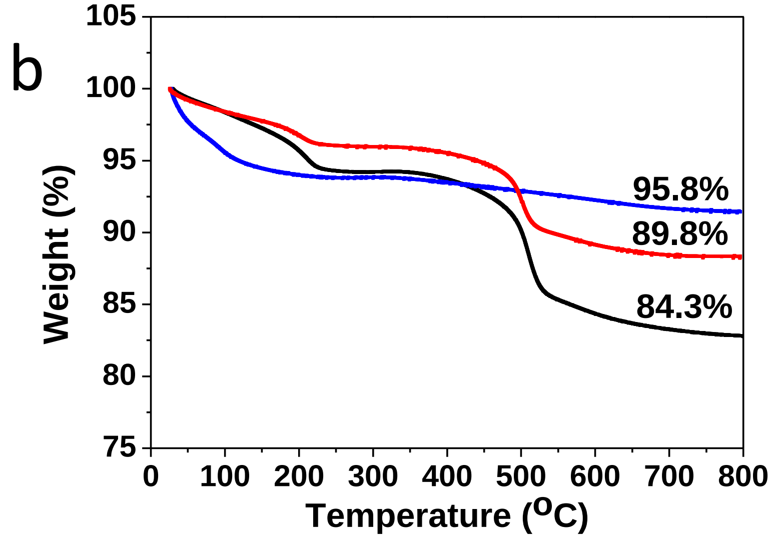

Figure S2: Thermogravimetric analyses of (a) Mn_2O_3/graphene and (b) $LiMn_2O_4$/graphene with various $I_{LMO:G}$ values.

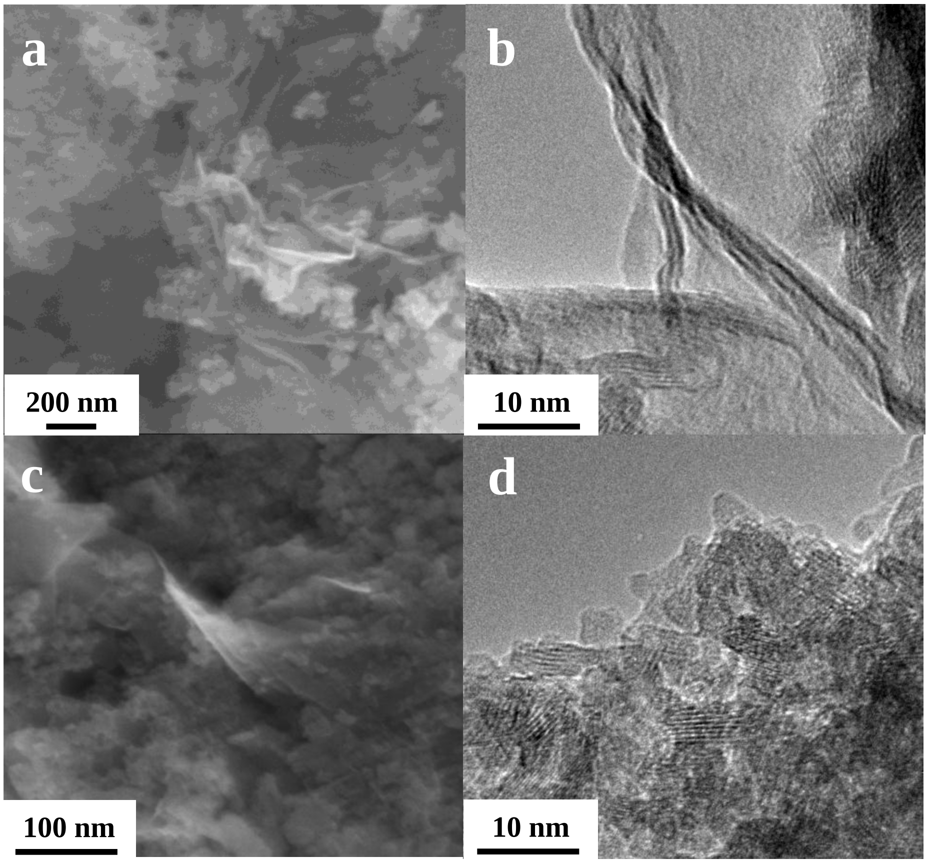

Figure S3: (a, c) SEM and (b, d) HRTEM images of $LiMn_2O_4$/graphene with (a, b) $I_{LMO:G}$ = 8.80 and (c, d) $I_{LMO:G}$ = 22.81.

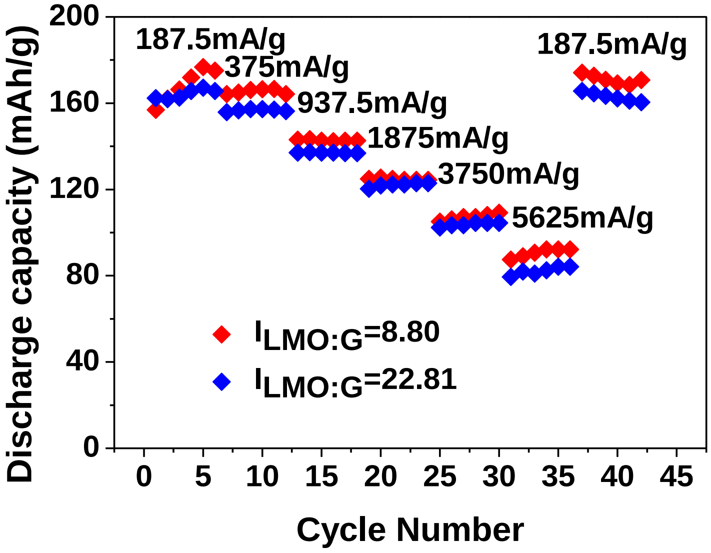

Figure S4: Rate performance of $LiMn_2O_4$/graphene with $I_{LMO:G}$ = 8.80 and 22.81 between 2 and 4.5 V.

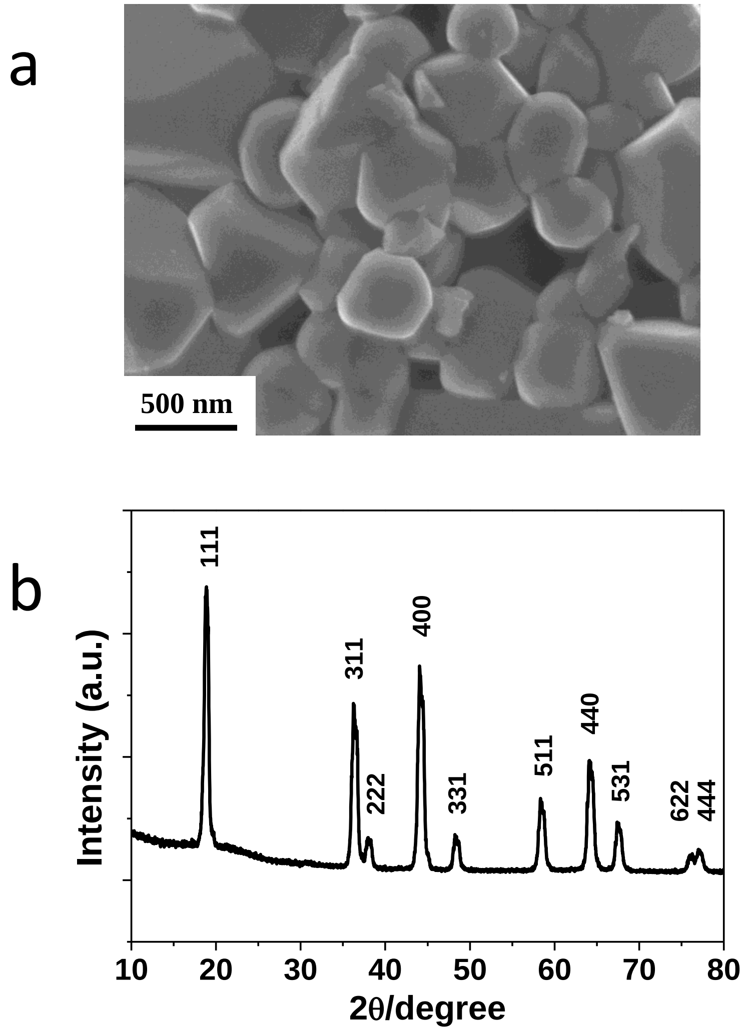

Figure S5: (a) SEM image and (b) XRD pattern of commercial $LiMn_2O_4$.

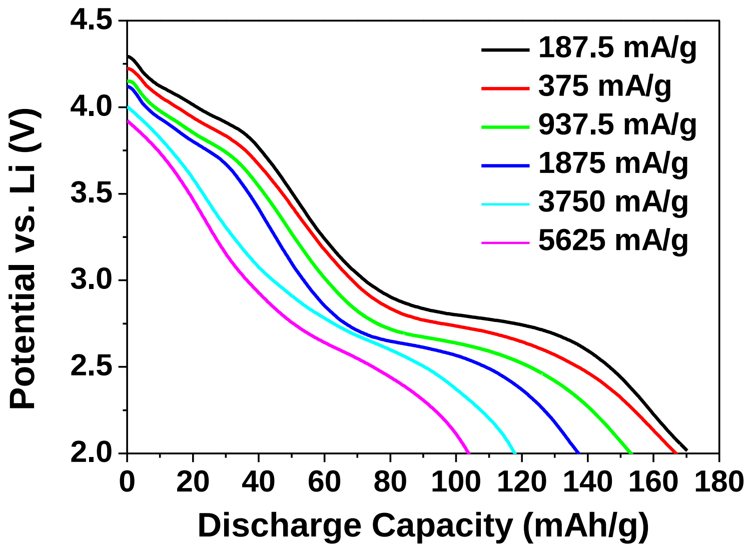

Figure S6: Discharge voltage profiles of LMO/G ($I_{LMO:G}$ = 5.37) electrode at various current densities.

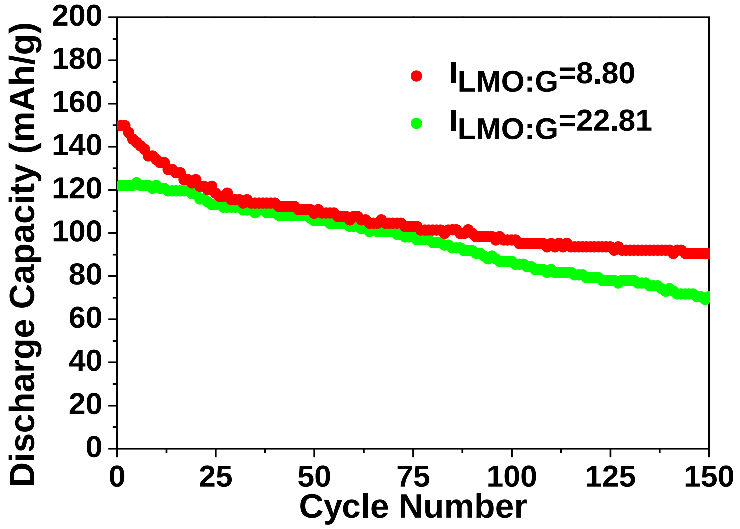

Figure S7: Cycling performance of $LiMn_2O_4$/graphene electrodes with different $I_{LMO:G}$ values at the 38 C discharge rate and 19 C charge rate.

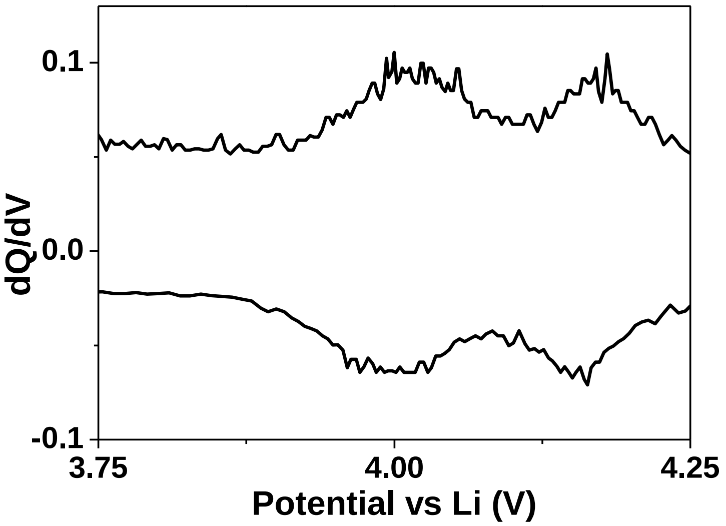

Figure S8: The curve of d*Q*/d*V* against potential (vs Li) of the second-cycle voltage profiles of $LiMn_2O_4$/graphene ($I_{LMO:G}$ = 1.22) between 3 and 4.5 V at 1.27 C.

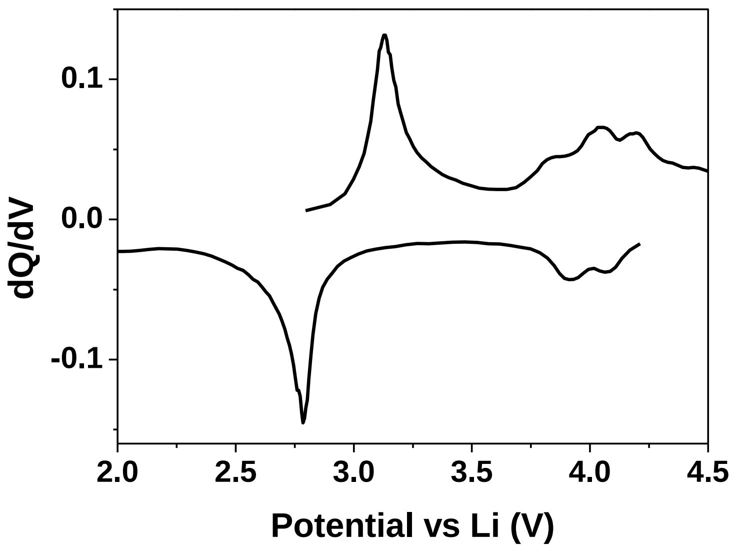

Figure S9: The curve of d*Q*/d*V* against potential (vs Li) of the second-cycle voltage profiles of $LiMn_2O_4$/graphene ($I_{LMO:G}$ = 1.22) between 2 and 4.5 V at 1.27 C.
